# Forensic isoscapes based on intra-individual temporal variation of *δ*^18^O and ^206^Pb/^207^Pb in human teeth

**DOI:** 10.1080/20961790.2020.1795377

**Published:** 2020-08-28

**Authors:** Laura A. Regan, Nathan W. Bower, Samuel J. Brown, Craig C. Lundstrom, Gideon Bartov, Matthew D. Cooney

**Affiliations:** a Office of Net Assessment, Department of Defense, Washington, DC, USA; b Chemistry and Biochemistry Department, Colorado College, Colorado Springs, CO, USA; c Geology Department, University of Illinois, Urbana, IL, USA; d Information Technology Services, Colorado College, Colorado Springs, CO, USA

**Keywords:** Forensic sciences, dentin, enamel, environmental, geolocation, isoscape, radiogenic and stable isotopes

## Abstract

Isotopic signatures used in the georeferencing of human remains are largely fixed by spatially distinct geologic and environmental processes. However, location-dependent temporal changes in these isotope ratios should also be considered when determining an individual’s provenance and/or trajectory. Distributions of the relevant isotopes can be impacted by predictable external factors such as climate change, delocalisation of food and water sources and changes in sources and uses of metals. Using Multi-Collector Inductively-Coupled Plasma Mass Spectrometer (MC-ICP-MS) analyses of ^206^Pb/^207^Pb in tooth enamel and dentin from a population of 21 ± 1-year-old individuals born circa 1984 and isotope ratio mass spectrometry (IRMS) of *δ*^18^O in their enamel, we examined the expected influence of some of these factors. The resulting adjustments to the geographic distribution of isotope ratios (isoscapes) found in tooth enamel and dentin may contain additional useful information for forensic identification, but the shifts in values can also impact the uncertainty and usefulness of identifications if they are not taken into account.

**KEY POINTS**
- Isoscapes of ^206^Pb/^207^Pb and *δ*^18^O used for geolocation are not static.
- Within a few years, the enamel and dentin of a person may exhibit measurable differences in ^206^Pb/^207^Pb even without changing locations.
- Changes in climatic patterns tied to rising temperatures are more significant than the direct effect of increasing temperature on *δ*^18^O fixed in tooth bioapatite.
- Third molar (M3) enamel mineralisation includes material incorporated from before formal amelogenesis takes place.

## Introduction

Isotopic analyses of human remains have proven valuable for forensic identification when more selective methods (e.g. dental records, fingerprinting or DNA analyses) cannot be used [1]. These analyses include light elements (e.g. carbon, nitrogen and oxygen: C, N and O) whose isotopes are fractionated due to their mass differences [2] and heavy elements (e.g. strontium and lead: Sr and Pb) that exhibit variation due to radiogenic differences.

Isoscapes (geographic distributions of isotope ratios plotted as topographies) for O and Pb measured in modern human teeth from United States Air Force Academy (USAFA) cadets (obtained from teeth collected by Regan in 2005/6 [3]), were recently developed by Keller et al. [4] for the conterminous USA. Empirical equations that related δ^18^O (δ defined below) and ^206^Pb/^207^Pb in the teeth to local surface water and aeolian dust proxies, respectively, were also developed [4]. Furthermore, their analyses of the inter- and intra-tooth and geolocation variations in that population for δ^13^C, δ^18^O, ^206^Pb/^207^Pb and ^87^Sr/^86^Sr found only δ^18^O and ^206^Pb/^207^Pb exhibited differences large enough for useful geolocation of individual cadets whose natal origins were within North America.

Small variations attributed to regional differences in wheat *versus* corn consumption were found in δ^13^C, and although ^87^Sr/^86^Sr was correlated with local tap waters, only about 20% of the variation could be attributed to those waters [4]. Sr is incorporated from food and water, so the poor link between ^87^Sr/^86^Sr and location in modern people’s teeth is likely due to delocalisation of civic water supplies post 1900, increasing use of bottled water and the rapid globalisation of food sources after World War II [5–7]. Isotopes of O and Pb that are incorporated in bioapatite are largely distributed by atmospheric processes, so broad regional patterns emerge in their isoscapes. For elements like C and Sr that are incorporated from food and water sources, high resolution or localized isoscapes are necessary in order to obtain useful geolocation information. Therefore, this study focuses only on O and Pb in modern individuals. Crown enamel and mid-root dentin Pb concentration and ^206^Pb/^207^Pb are mapped, and the increasing use of recycled Pb on these is examined. Variations in δ^18^O due to climate fluctuations recorded in the enamel are also explored and expected differences between the enamel and dentin, structural carbonate δ^18^O_c_ values are modelled.

Ratios for light isotopes such as ^18^O/^16^O are usually measured *versus* a standard such as the Vienna Pee Dee Belemnite (VPDB) and expressed in delta (δ) notation in parts per thousand:

(1) $$\delta^{18}O_{\text{VPDB sample}}\;=\;\{\frac{\left[{}^{18}O/{}^{16}O{)}_{\mathrm{sample}}\right]}{\left[({}^{18}O/{}^{16}O{)}_{\mathrm{VPDB}}\right]}-1\}\times 1000‰.$$

Conversion between VPDB and Vienna Standard Mean Ocean Water (VSMOW) δ^18^O_w_ values can be made with a relationship such as Coplen et al.’s [8]:

(2) $$\delta^{18}O_{w(\text{VSMOW})}=\;1.0392\;\times \;\delta^{18}O_{w(\text{VPDB})}+\;3.092‰.$$

Temporal variations in δ^18^O and ^206^Pb/^207^Pb based on sediment or fossil records that span centuries or millennia have been published [4,9–12], but recently attention has begun to focus on temporal changes in a given location that can impact assumed geolocation trajectories recorded within individuals. Kennedy et al. [13] measured the monthly variation of δ^18^O in ground and surface drinking waters in 2005 and 2006 and used the data to construct isoscapes for drinking water in the conterminous USA. The spatiotemporal variations were used to successfully track the two-year trajectory of an individual of forensic interest based on oxygen isotope ratios in a 26 cm hair sample. Both seasonal fluctuations and geolocation differences were important.

While Kennedy et al.’s study [13] resolved many questions about the temporal impact of climatic changes on human δ^18^O trajectories, more research is needed to determine the time-dependent effects on Pb and its isotopes. Just like the trajectories recorded in hair, human bioapatite incorporates the isotopic profiles of where an individual has been, but the time scale is measured in years instead of months. Tracing these trajectories is only feasible if the intra-location variation is smaller than the inter-location variation. Using micro-sample methods such as laser ablation (LA) and secondary-ion mass spectrometry (SIMS) [14], sequential layers in enamel have been probed to obtain time-dependent profiles of C, O, Ba, Pb, Sr and Zn [15]. However, a number of potential problems have been identified, especially for δ^18^O analyses. For example, bioapatite has O bound in its organic, PO_4_, CO_3_ and OH components, each with different mobilities and metabolic pathways [15–20]. Furthermore, enamel and dentin form in at least two stages: a matrix formation stage with some mineralisation, and then a maturation stage during which most mineralisation occurs. These two stages have various cycles and rates for different elements in multiple growth directions [15,21]. The resulting heterogeneity can cause inconsistencies in results if insufficient sample sizes and different parts of the tooth tissues are analysed.

This study focuses on quantifying the differences (Δ*s*) in Pb concentrations and Pb isotope ratios found in same-tooth (third molar, M3) crown enamel versus mid-root, primary dentin from a population of similar-age individuals who grew up in single locations distributed across the conterminous USA. Spatiotemporal changes in δ^18^O_c_ are explored by examining the variation in δ^18^O_c_ for an additional population of USAFA cadets who changed locations at different times. Adjustments to the temporal changes are modelled using National Oceanic and Atmospheric Administration/National Centres for Environmental Information (NOAA/NCEI) data for multi-year surface temperature anomalies converted to δ^18^O_c_ values [22]. Temporal changes in the cadets’ Pb and O tooth isotope ratios are compared to isoscapes for the mean year (1996) for enamel formation to determine how important these changes are to geolocation trajectories.

The mineral portion of the bioapatite in tooth enamel is a carbonate-substituted hydroxyapatite (Ca_10_(PO_4_)_6 –_ *_x_*(OH)_2 –_ *_y_*)(CO_3_)*_x_*_+_ *_y_* that comprises more than 95% by mass of the enamel, with less carbonate at the surface [23]. Trace amounts of Pb (primarily ingested from aeolian-distributed dust deposition [3,24]) substitute for Ca in the bioapatite formed during amelogenesis (enamel formation). Most M3 enamel mineralisation begins after age 8 and is complete by age 16. It tends to be later (ca. 0.5 years) in males [25,26], but this difference is negligible compared to the age range. Primary dentin formation in M3 also fixes Pb, but the root mineralizes from the crown and reaches maturity between age 18 and 25 [27,28]. While enamel has virtually no natural turnover once amelogenesis is complete, Pb turnover rates of about 1%–3%/year have been measured in dentin [24,29,30]. Thus, differences between enamel and mid-root dentin found in M3 extracted in 2005/6 from circa 21-year-old individuals born circa 1984 and who resided in single locations, should be useful for measuring integrated changes in local isotope ratios during the 6-year interval between enamel (age 12 ± 2) and mid-root dentin (age 18 ± 2) mineralisation [27,28].

As noted above, predicted differences in the isotope ratios for O and Pb between enamel and dentin arise for different reasons. Oxygen’s isotopes are incorporated in humans primarily from imbibed drinking water and beverages [31]. Water is fractionated during both evaporation (lighter molecules escape) and precipitation (heavier molecules are removed). These are temperature dependent processes, and to a first approximation, higher elevations and higher latitudes are more depleted (more negative δ^18^O) in the heavier ^18^O isotope relative to VSMOW. Average surface temperatures in the conterminous USA have been rising about 0.025 °C/year since 1980 [22], and these increases should shift δ^18^O_c_ towards more ^18^O (less negative δ^18^O values) by about 0.5‰/°C, although these shifts will be different in different locations in response to changes in factors such as wind direction, water source and precipitation temperatures [32].

While O is taken up from natural sources, Pb is incorporated primarily from anthropogenic sources. ^204^Pb is constant while ^206^Pb, ^207^Pb and ^208^Pb accumulate from the decay of ^238^U, ^235^U and ^232^Th, respectively. Thus, geological ores develop characteristic isotopic ratios over millions of years and humans incorporate Pb during environmental exposure. Values for ^206^Pb/^207^Pb found in batteries, bullets, pigments, solder and gasoline additives vary from 1.04 in Northern Australia to 1.47 for some Upper Mississippi Valley Type (MVT) ores [33,34].

Most people incorporate Pb from ingestion of Pb distributed via aeolian dust, as Pb from the combustion of gasoline and fumes released from smelters oxidizes in the atmosphere and is removed by wet and dry deposition. Most of this removal takes place within tens to hundreds of kilometres depending on factors such as wind speed, direction, the nature of the surface, the size of the particles formed and precipitation levels [35]. However, very small particles are also transported globally [36]. Crawling children who lick their hands directly ingest dust from old paint, aeolian dust, etc., while adults incorporate Pb more from smoking, water and food sources [37].

Natural processes mix Pb released to the environment, but mixing of Pb from different sources through industrial recycling of the metal is likely a more important process in the USA for individuals born circa 1984. Secondary (recycled) domestic Pb production was near 28% in 1928, 55% in 1985, 86% in 2005 and has been near 100% since 2013 when the last primary Pb smelter in the USA was closed [38]. This amounts to an average growth rate of 1.5% recycled Pb/year during the period of this study. While the proportion of Pb from secondary sources has been increasing, total production has also been increasing and during the 1990s, the relative amount from the MVT sources in Missouri grew [38], suggesting that Pb released to the environment, especially in the Midwest and around the Great Lakes (downwind from Missouri), should exhibit a small increase in ^206^Pb/^207^Pb values relative to declines in the rest of the country. These changes are of interest for developing more nuanced forensic isoscapes.

## Methods

Before collecting and using samples from live subjects, appropriate permissions were obtained. (Documentation may be found in Regan [3].) Only M3 (crown completion circa 1996–1999; root completion 1996–2005) extracted during the winter of 2005/6 from USAFA cadets born in 1984 ± 1 (1 s) were used in this study. Two outliers born in 1979 were included in the Pb study for statistical comparison. Thus, dentin remodelling was negligible. Molars used to develop the isoscapes in this study were from individuals who self-reported living in a single location from their year of birth until after 2000. The sex and race/ethnicity demographic profile of the samples used for the isoscapes were based on self-identification: 80% male, 20% female, 88% White, 6% Hispanic (any race), 4.5% Black/African and 1.5% Asian [3].

Regan’s analyses of the molars’ enamel for ^206^Pb/^207^Pb (*n* = 23) and δ^18^O_c_ (*n* = 158) were described previously [3,4]. Most of these individuals remained in a fixed location between ages 8 and 18. Excluding those born before 1981 or who lived in multiple locations, left *n* = 90 individuals for developing a fixed-in-time, 1996, δ^18^O_c_ isoscape. An additional 28 individuals who relocated once before age 8 were also identified. Their deviations from isoscape predictions were used to test the δ^18^O_c_ isoscape. (Regan’s δ^18^O_c_ enamel data are reproduced in Table S1 of the Supporting Information.)

All teeth used in Regan’s [3,4] and this study were first cleaned of organic matter and sterilized by soaking the teeth in 3% H_2_O_2_ for 2 days. Any adherent periodontal tissue was also removed. After sonicating in 18 MΩ water for 30 min and subsequent drying, interior enamel powder was obtained using a carbide drill bit. The powders were subsequently treated with 30% H_2_O_2_ to remove organic residues and dilute acetic acid to remove any secondary carbonates before analysis [3].

In Regan’s study, the enamel powder analysed for Pb was dissolved overnight in 8 mol/L HNO_3_ (triply distilled) in Teflon vials and taken to dryness. Pb bromide was formed by dissolving the residue with 1 mol/L HBr (Optima) and separated in a class-1000 clean lab using Dowex 1X-8 micro-columns following the method of Manhes et al. [39]. Analyses of the Pb isotope ratios were conducted using a Nu-Plasma, Multi-Collector Inductively-Coupled Plasma Mass Spectrometer (MC-ICP-MS). The enamel’s structural carbonate δ^18^O_c_ was measured in the enamel powder using a VG/Micromass PRISM Series II isotope ratio mass spectrometer (IRMS) with 90 °C H_3_PO_4_ in an automatic Isocarb preparation device. (The H_3_PO_4_ is used to convert the enamel carbonates to CO_2(g)_. Note that different acid temperatures can create measurable isotope fractionations [40].)

To develop more detailed Pb isoscapes and to measure the circa 6-year difference between the cadets’ crown enamel and mid-root, primary dentin, *n* = 43 additional M3 not analysed in the previous study were prepared and analysed for the enamel’s Pb concentration and ^206^Pb/^207^Pb using a procedure based on Keller et al. [4], outlined below. Because of funding limitations, only a subset of these (*n* = 16) were ultimately analysed for their dentin’s Pb concentration and Pb isotopes. These were selected to maximise the coverage of the conterminous USA so that at least regional differences might be detected. Molars analysed for Pb were also selected that replicated sample locations in Regan’s [3] investigation in order to cross-validate the studies. Both this study and Regan’s [3] included analyses of laboratory blanks and NIST standards.

In this study, tooth surfaces were cleaned with a carbide burr and sonication in 18 MΩ H_2_O that removed any remaining surface contamination and tissues. Crown enamel and mid-root dentin samples (10–20 mg) were removed inside of virgin polypropylene (PP) bags to avoid contamination. Dentin and enamel were manually selected under magnification. Following the method in Bower et al. [41] and Keller et al. [4], these samples were dissolved overnight in 1.0 mL of 16 mol/L triply distilled HNO_3_ in capped, virgin PP vials on an orbital shaker at 200 rpm. The solutions were diluted to 5.0 mL with 18 MΩ H_2_O, 20 mg of Eichrom Pb resin was added and the mixture was placed on an orbital shaker at 200 rpm for 1 h. Cleaning of the resin-bound Pb was performed by capturing the resin in acid-washed and rinsed PTFE columns followed by 3.0 mL of 2.0 mol/L HNO_3_. Extraction of the Pb was achieved with 3.0 mL 18 MΩ H_2_O and 1.0 mL 0.050 mol/L ammonium oxalate (Sigma, ≤0.01% trace metals) collected in PP vials which had 1.0 mL of 2.0 mol/L HNO_3_ added to avoid subsequent Pb precipitation.

The Pb solutions were analysed for their Pb concentrations using a Thermo-Fisher iCAP Q ICP-MS. The data were collected with an instrumental precision of about 5% and a detection limit of 0.03 ppb. Pb isotope ratios were obtained using a Nu-Plasma High Resolution MC-ICP-MS. A Tl spike used for mass-bias correction was added to each sample just before analysis [33,42] and sample bracketing with NIST-981 was used to correct for any instrument drift.

We predicted temperature fluctuations over time would create deviations from the measured 1996 δ^18^O_c_ isoscape. These Δδ^18^O_c_ offsets before and after 1996 were modelled using historic records of annual temperature anomalies [22]. These were used to estimate the magnitude and sign that changing locations should have on an individual’s enamel δ^18^O_c_. These temperature changes were also used to calculate the offset expected between the median time of crown formation (1996) and mid-root formation (2002) [28].

To create an isoscape of these differences, the Δ°C anomalies for the NOAA/NCEI US Climate Divisions were used to estimate corresponding Δδ^18^O_c_ values by subtracting the Bayesian-weighted data for 1996 from the data for 2002 in each climate division [43]) and then using Equations (3) and (4) (see below) to convert Δ°C to Δδ^18^O_c(VPDB)_. For simplicity, the δ^18^O_w_ to δ^18^O_c_ conversions were assumed the same for enamel and dentin and the Bayesian weights were assumed to follow a 5-year square distribution centred on 1996 and 2002, respectively. Because mineralisation occurs in episodic cycles that span multiple years, a square distribution was thought to provide as realistic a weighting of the fluctuations as a Gaussian distribution. Only interior enamel was used for the analyses, narrowing the time range [15,28].

(3) $$\Delta \delta^{18}O_{w(\text{VSMOW})}=\;\alpha \;\times \;\Delta {}^{\circ}C$$

(4) $$\Delta \delta^{18}O_{c(\text{VPDB})}=\;0.47\;\times \;\Delta \delta^{18}O_{w(\text{VSMOW})}$$

The Rayleigh equation for isotope fractionation at equilibrium produces a curved relationship with smaller slopes at higher temperatures [44]. However, temperature increments (Δ°C) of a few degrees may be fit with a linear model for the dependence of δ^18^O_w_ on surface temperature. Values for a nonequilibrium *α* in Equation (3) depend on factors such as the type of precipitation (snow *vs*. rain), the temperature of the source water, the condensation temperature and how much of the original water vapour has already precipitated out. Using global mid-latitude data, Fricke and O'Neil [45] obtained an *α* = 0.578 for the winter and 0.417 for the summer. Liu et al. [46] measured a winter *α* = 0.64 and a summer *α* = 0.38 for 1992 and 0.63 and 0.56, respectively, for 1993 for the USA. Dansgaard [32] gave a number of different values for *α* around 20 °C ranging from 0.24 to 0.78 δ^18^O‰/°C for various conditions. Thus, +0.5 δ^18^O‰/°C was used for estimating the responsivity of δ^18^O_w(VSMOW)_ to temperature in Equation (3), as our method of sampling the enamel and dentin included multiple years that average the *α* extremes. Fast-growing tissues, such as hair [13], give a better analysis of seasonal variations [47], but species differences in the secretion and maturation cycles in tooth formation are still being determined [48]. Equation (4) (obtained by rearranging Equation (5) in Keller et al. [4]) was then used to estimate the conversion of Δδ^18^O_w(VSMOW)_ to Δδ^18^O_c(VPDB)_.

The data were statistically analysed using Microsoft Excel 2016 and Minitab ver.18.1 (Minitab, Inc., State College, PA, USA). Isoscapes for the Pb and O data were plotted using ArcGIS version 10.4.1 with Spatial Analyst Extension (Esri, ArcGIS, Redlands, CA, USA). Interpolation between points used an inverse square of the distance for the weighting of points, and contours were extended to the USA boundaries and then clipped.

## Results and discussion

**Figure 1.**
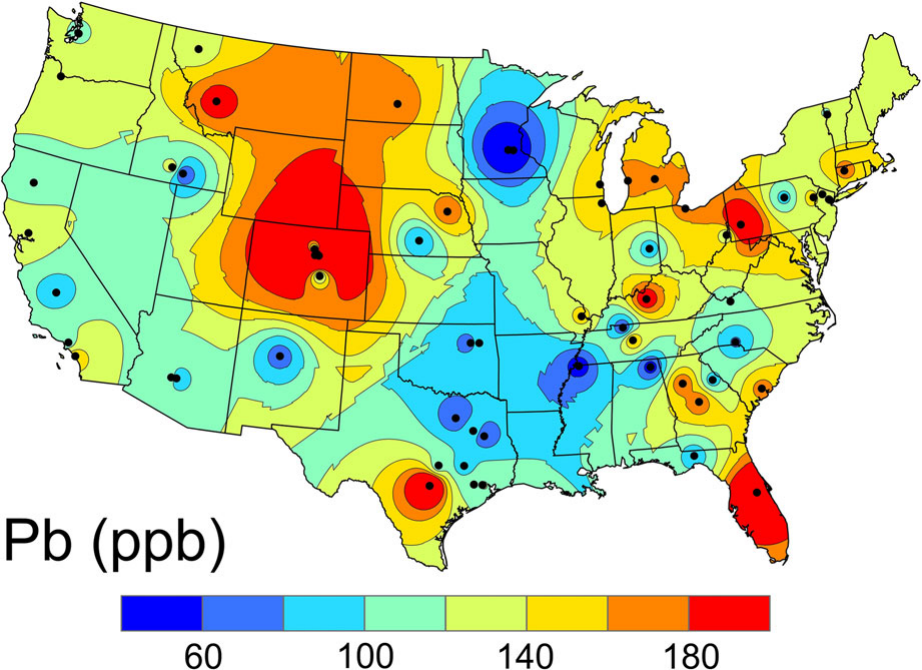
Map of the Pb concentration in the United States Air Force Academy (USAFA) cadets’ crown enamel (formed ca. 1996).

The Pb concentrations and isotope ratios from the 43 individuals measured during this study are presented in Table 1. Data from the 23 individuals previously analysed by Regan [3] are presented in Table 2. Instrument errors and laboratory blanks for the ^206^Pb/^207^Pb data (Table 1) were negligible for these common-Pb samples. Figure 1 shows the enamel-based Pb concentration isoscape obtained from the individuals with a known and consistent geolocation during amelogenesis and dentinogenesis. Although the pattern is somewhat similar to that expected for oxygen (see below), the highest concentrations of Pb are primarily found downwind in an easterly direction from historic Pb-emission sites (e.g. smelters, urban traffic and coal burning) rather than being coupled to Pb-mine sources.

**Table 1.** Locations and Pb concentration and isotope data from the United States Air Force Academy (USAFA) cadets' third molar crown enamel and mid-root dentin from individuals who lived in the same location from the year of birth (ca. 1984) through amelogenesis (ca. 1996).

			Enamel					Dentin				
Identity	Latitude	Longitude	208/204	207/204	206/204	206/207	Pb (ppb)	208/204	207/204	206/204	206/207	Pb (ppb)
AFA-013	35.085	–106.606	38.502	15.661	18.904	1.2073	71	38.614	15.728	19.015	1.2090	230
AFA-028	29.786	–95.824	38.496	15.733	18.829	1.1977	105					
AFA-029	29.763	–95.383	38.600	15.711	18.923	1.2045	123					
AFA-034	47.606	–122.332	38.912	15.809	18.915	1.1965	119	38.448	15.689	18.799	1.1982	105
AFA-048	29.703	–98.125	38.279	15.628	18.621	1.1916	277	38.159	15.632	18.463	1.1813	255
AFA-059	43.013	–87.973	38.493	15.655	18.888	1.2066	153	38.446	15.647	18.831	1.2036	162
AFA-073	32.205	–95.856	37.785	15.629	17.996	1.1526	97					
AFA-080	41.512	–82.938	38.524	15.672	18.842	1.2025	165					
AFA-082	42.061	–93.886	38.721	15.722	19.123	1.2164						
AFA-083	31.964	–95.269	38.598	15.704	18.943	1.2062	59					
AFA-094	33.019	–80.176	38.424	15.653	18.784	1.2002	166	38.365	15.635	18.711	1.1968	204
AFA-097	30.628	–96.334	38.470	15.680	18.795	1.1981	80	38.511	15.676	18.889	1.2059	283
AFA-102	41.034	–74.636	38.355	15.628	18.682	1.1961	117					
AFA-105	40.748	–74.323	38.402	15.673	18.768	1.1976	143					
AFA-115	35.925	–86.869	38.281	15.647	18.597	1.1888	160	38.377	15.652	18.765	1.199	279
AFA-119^a,b^	44.944	–72.204	38.423	15.638	18.811	1.2027						
AFA-136	42.725	–114.518	38.559	15.683	18.937	1.2073	130					
AFA-138	35.262	–81.187	38.532	15.682	18.854	1.2024	77	38.331	15.639	18.582	1.1884	180
AFA-149	37.180	–89.655	38.626	15.700	19.046	1.2132	146					
AFA-157	42.033	–97.414	38.404	15.634	18.818	1.2043	174					
AFA-166	46.808	–100.784	38.218	15.625	18.525	1.1859	169	38.416	15.648	18.729	1.1970	177
AFA-168	40.421	–79.788	38.274	15.651	18.621	1.1900	265					
AFA-171	40.719	–74.285	38.361	15.657	18.688	1.1938	126	38.254	15.64	18.581	1.1882	252
AFA-182	40.987	–75.195	38.390	15.654	18.668	1.1927	152					
AFA-185	36.098	–119.56	38.496	15.682	18.846	1.2017	88	38.379	15.648	18.722	1.1967	152
AFA-186	36.151	–95.509	38.445	15.668	18.834	1.2021	98					
AFA-192	45.523	–122.677	38.421	15.650	18.802	1.2015	130	38.324	15.639	18.675	1.1942	168
AFA-197	43.001	–84.559	38.416	15.678	18.766	1.1974	161					
AFA-199	32.776	–96.797	38.509	15.697	18.858	1.2014	58					
AFA-201	39.929	–85.370	38.828	15.742	19.122	1.2149	90					
AFA-203	44.768	–93.278	38.577	15.739	18.909	1.2011	63	38.521	15.696	18.894	1.2037	82
AFA-205	33.749	–84.388	38.468	15.669	18.814	1.2009	179					
AFA-212	46.004	–112.535	38.367	15.642	18.685	1.1947	193					158
AFA-222	36.154	–95.993	38.133	15.633	18.464	1.1817	72					
AFA-223	33.523	–117.708	38.335	15.644	18.578	1.1877	154	38.41	15.665	18.689	1.1934	87
AFA-224	40.015	–105.271	38.550	15.740	18.834	1.1962	81	38.291	15.636	18.572	1.1879	186
AFA-228	40.064	–80.721	38.335	15.640	18.744	1.1985	126					
AFA-231	43.063	–86.228	38.372	15.657	18.720	1.1958	173					
AFA-246	48.192	–114.317	38.450	15.677	18.802	1.1993	132	38.267	15.630	18.610	1.1909	160
AFA-250	34.052	–118.244	38.468	15.672	18.723	1.1948	106					
AFA-257	41.254	–76.921	38.449	15.672	18.756	1.1959	92					
AFA-265^a^	38.834	–104.821	38.492	15.656	18.866	1.2052	111					
AFA-272	32.841	–83.632	38.426	15.659	18.807	1.2012	178					
Ave. blank			38.636	15.551	18.535	1.1918	0.76					
1s error			0.004	0.002	0.002	0.0002	5%^c^					
NIST-981			36.7018	15.4904	16.9379	1.0935						
981, 1s			0.0047	0.0010	0.0011	0.0001						
Cert. value			36.7219	15.4916	16.9374	1.0933						

^a^Year of birth outlier data.

^b^Moved within upper Vermont.

^c^Percent relative standard deviation.

**Table 2.** Original enamel data from Regan [3] used with the data in Table 1 to construct the ^206^Pb/^207^Pb isoscape. The ^206^Pb/^207^Pb was obtained by dividing the ^206^Pb/^204^Pb data by the ^207^Pb/^204^Pb data.

Identity	City	State	208/204	207/204	206/204	Pb (ppm)
AFA-004	Clarksville	TN	38.4127	15.6477	18.8067	0.06
AFA-006	Laguna Niguel	CA	38.3241	15.6381	18.6474	0.14
AFA-017	Phoenix	AZ	38.4275	15.6492	18.8189	0.09
AFA-021	Goodyear	AZ	38.3369	15.6418	18.6627	0.11
AFA-031	Denver	CO	38.3722	15.6484	18.8110	0.43
AFA-032	Sprucegrove	Alberta	38.2715	15.6369	18.6870	0.15
AFA-051	Arvada	CO	38.3673	15.6436	18.7789	0.14
AFA-056	Chaska	MN	38.3927	15.6595	18.7845	0.02
AFA-060	Kearney	NE	38.6165	15.6749	19.0493	0.08
AFA-078	Burlington	VT	38.3427	15.6374	18.6884	0.09
AFA-085	Torrington	CT	38.2804	15.6291	18.5967	0.17
AFA-089	Orlando	FL	38.3107	15.6324	18.6480	0.24
AFA-096	Bland	VA	38.3654	15.6432	18.7485	0.11
AFA-103	Golden	CO	38.2570	15.6279	18.6099	0.32
AFA-111	Old Shasta	CA	38.2731	15.6260	18.6255	0.11
AFA-133	Scottsboro	AL	38.2865	15.6256	18.6444	0.05
AFA-134	Southaven	MS	38.4202	15.6564	18.8606	0.05
AFA-146	Burley	ID	38.2489	15.6265	18.5676	0.06
AFA-163	Houston	TX	38.4059	15.6306	18.6732	0.12
AFA-164	Washington	GA	38.3386	15.6357	18.7481	0.09
AFA-173	Vacaville	CA	38.1825	15.6251	18.4710	0.14
AFA-174	Elizabethtown	KY	38.3703	15.6463	18.7275	0.2
AFA-176	Tallahassee	FL	38.3703	15.6431	18.7920	0.09
Blank						<0.01
NIST-981			36.695	15.490	16.937	
981,1s			0.009	0.003	0.004	

**Figure 2.**
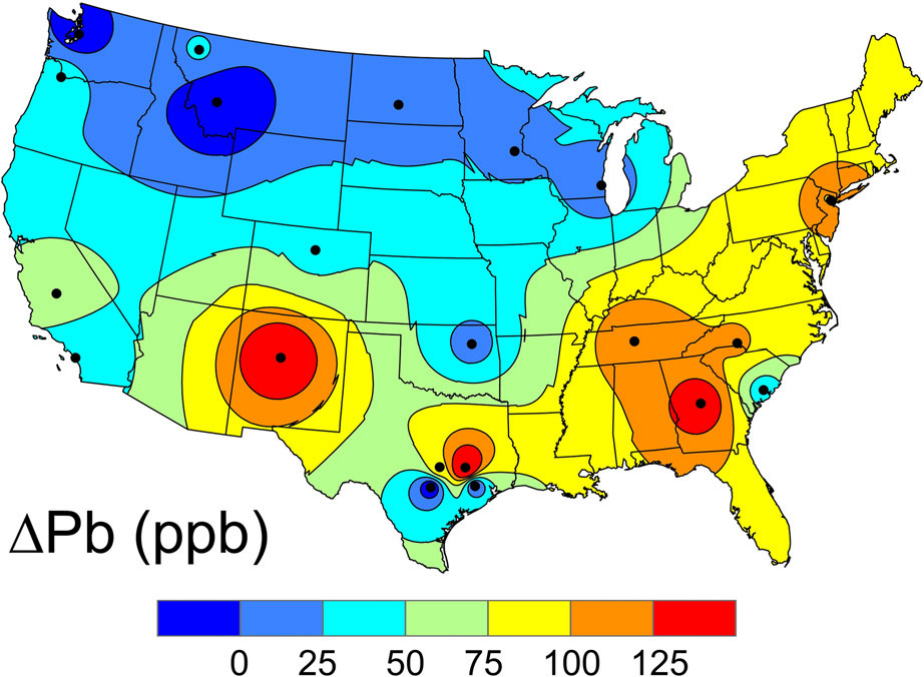
Map of the shift in the Pb concentration (ΔPb) in the United States Air Force Academy (USAFA) cadets’ mid-root dentin (formed ca. 2002) compared to their crown enamel (formed ca. 1996).

The data in Figure 2 illustrate the change in the spatial variation of the concentration of incorporated Pb (a ΔPb map, obtained by subtracting the enamel Pb from the dentin Pb) in the conterminous USA. In combination with Figure 1, the data in Figure 2 suggest individuals in the southern states started with lower levels of Pb, but these levels are increasing faster than in the Northern Plains and Rocky Mountain States. Some of this increase may be due to increasing USA Pb production, which went up by 37 500 tonnes/year during the 1980s and 1990s, a 79% increase between 1984 and 2005 [38]. The 43% ± 11% higher average in the dentin versus the enamel is readily explained by this growth in production. Furthermore, more secondary Pb smelters are located in the southern and eastern states and more were relocated to northern Mexico, which had much lower emission standards. These emissions are carried by seasonal winds into the southeastern states [49]. Although blood lead levels (BLLs) have been declining in the USA overall, lower income areas with older infrastructure and homes with decaying Pb paint as well as higher population densities have not experienced the same amount of improvement [50].

**Figure 3.**
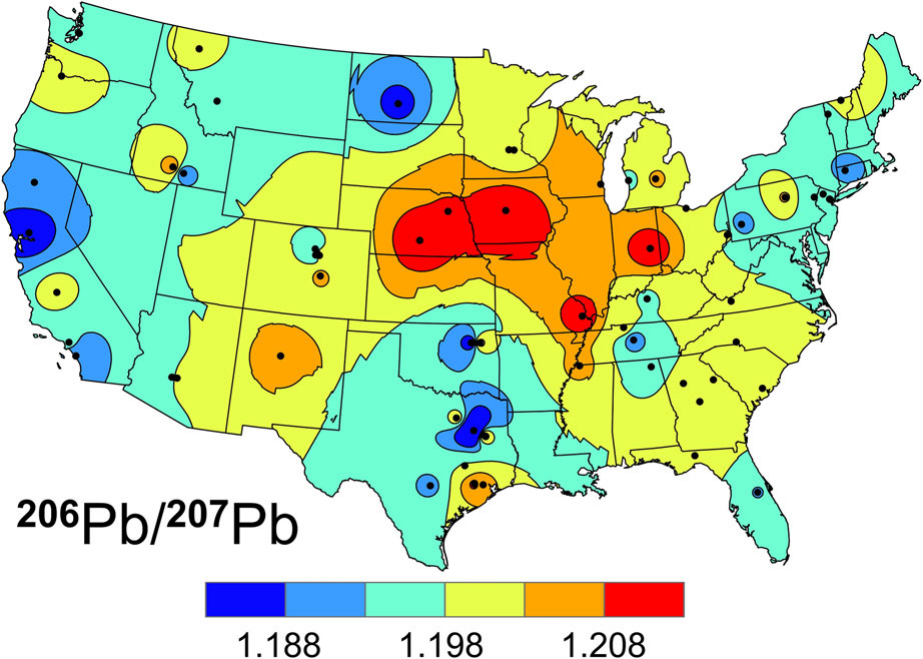
Isoscape of the ^206^Pb/^207^Pb in the United States Air Force Academy (USAFA) cadets’ crown enamel (formed ca. 1996).

Figure 3 is an isoscape of ^206^Pb/^207^Pb in the USAFA cadets’ tooth enamel. (Other ratios, such as ^208^Pb/^206^Pb, produce isoscapes with similar patterns: not shown.) A ratio of 1.19 for ^206^Pb/^207^Pb is typical of background levels in soils, while values above 1.20 are indicative of more recently formed Pb ores. Gasoline made from Upper Mississippi Valley Type Pb exhibits ratios above 1.20 [51]. Lead ores imported from South America and Mexico as well as Pb mined in Idaho tend to have ^206^Pb/^207^Pb values below 1.18 [41], and this may be seen in the lower ratios found along the west coast and in southern and western Texas where these ores were historically smelted. Furthermore, trans-Pacific airborne dust may also contribute, as Australia has been a major supplier of Pb to East Asia, and important ore sources there have ^206^Pb/^207^Pb values circa 1.04 [33].

**Figure 4.**
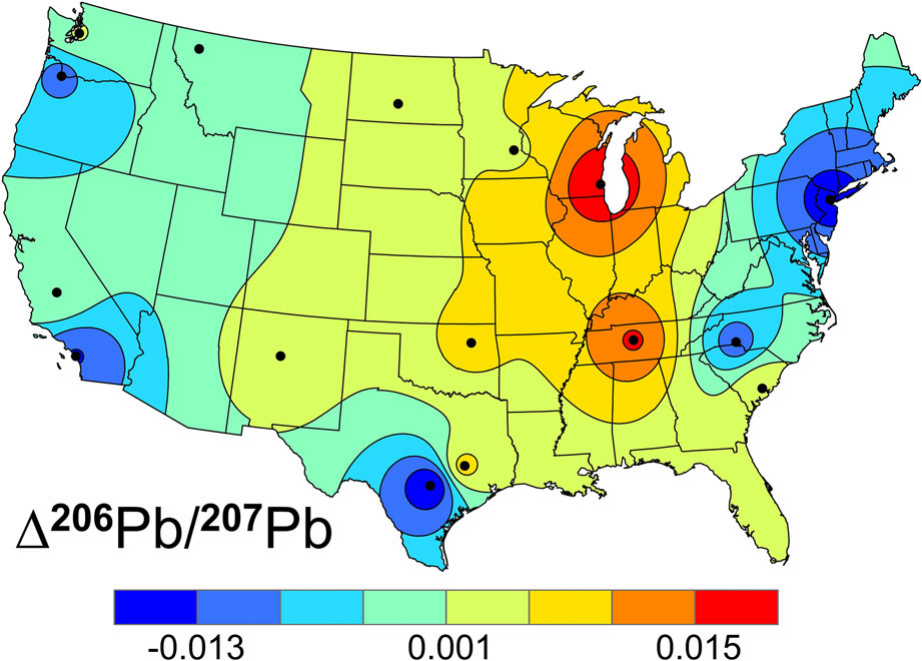
Isoscape of the shift in the ^206^Pb/^207^Pb (Δ^206^Pb/^207^Pb) in the United States Air Force Academy (USAFA) cadets’ mid-root dentin (formed ca. 2002) compared to their crown enamel (formed ca. 1996).

In Figure 4, the differences (Δ^206^Pb/^207^Pb) in ^206^Pb/^207^Pb in the cadets’ enamel and dentin are plotted. Isotopes ^204^Pb, ^206^Pb, ^207^Pb and ^208^Pb are too heavy to be fractionated by metabolic processes, so changes in the ratios indicate changes in the Pb sources between circa 1996 and 2002. Although leaded gasoline was largely phased out between 1976 and 1996 [52], the layer of dust it left as a legacy remains a source of background Pb that is slowly being mineralized and buried. Differences downwind from primary ore smelters in eastern Nebraska, Missouri and western Texas are probably more important than the background gasoline signature, as these locations also fit with the expected high and low isotope ratios, respectively. This can be seen in the (dentin–enamel) isoscape shown in Figure 4. Although more samples are needed to fill in the Δ^206^Pb/^207^Pb isoscape and more work can be done using bi-plots of isotopic ratios (e.g. ^208^Pb/^204^Pb *vs.* ^206^Pb/^207^Pb) to better isolate the sources of Pb in specific areas, the differences are in keeping with expectations from wind patterns and source mixing. Furthermore, both the concentration (±64 ppb, 1 s) and isotope (±0.009 ^206^Pb/^207^Pb, 1 s) data and their corresponding Δ data exhibit larger variations than the measurement errors (±5% of the concentration values, and ±0.002 ^206^Pb/^207^Pb, respectively).

Because the Δ plots are calculated from the differences of two values, their measurement errors will be at least $\sqrt{2}$ times larger. Model-interpolation errors (e.g. ±0.004 ^206^Pb/^207^Pb for Figure 3) are more problematic, especially in regions far from any measurements. The magnitude of the errors depend on the distribution and number of points used to construct the plots. Still, the Δ plots contain useful information. They show that the regional dentin profiles mimic the enamel profiles, but with some additional, time-related differences.

**Figure 5.**
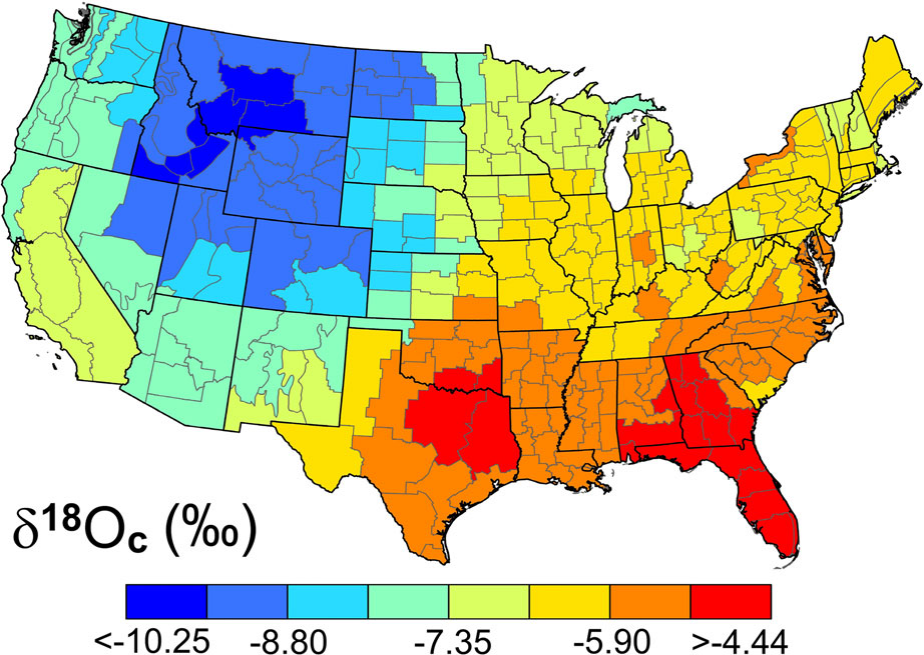
Isoscape of the δ^18^O_c(VPDB)_ in the United States Air Force Academy (USAFA) cadets’ crown enamel (formed ca. 1996) with an interpolated average plotted for each National Oceanic and Atmospheric Administration/National Centers for Environmental Information (NOAA/NCEI) climate division.

Data for δ^18^O_c(VPDB)_ measured by Regan [3] (used without conversion to δ^18^O_c(VSMOW)_ or correction for any method offsets) were used to construct Figure 5. Assuming an average time for mineralisation during amelogenesis of 12 years and a 1984 ± 1 birth year, Figure 5 is a plot of the δ^18^O_c_ incorporated into the crown enamel by these individuals ca. 1994 − 1998. Keller et al. [4] plotted the δ^18^O_c_ enamel data for a larger group of the USAFA cadets that included multiple birth years and a few who had changed locations. That enamel data were significantly correlated with the circa 1984 surface water data from Coplen and Kendall (Pearsonian *R*^2^ = 0.81, *P* < 0.0001, *df* = 154) [43]. The δ^18^O_c(VPDB)_ data for the essentially same-location, same-age individuals in Figure 5 are significantly correlated with Bowen et al.’s circa 2005 tap water data [53]: δ^18^O_c(VPDB)_ = −4.54 ± 0.25 + 0.345 ± 0.028×(δ^18^O_tap water(VSMOW)_); *R*^2^ = 0.75, *P* < 0.001, *df* = 50, ±1SE). Gaps in sample locations between the tooth and tap water data as well as disparities in tap water sources (ground, surface or managed) and residence times (0 to 100 years) explain some of the differences in the isoscapes [13].

**Figure 6.**
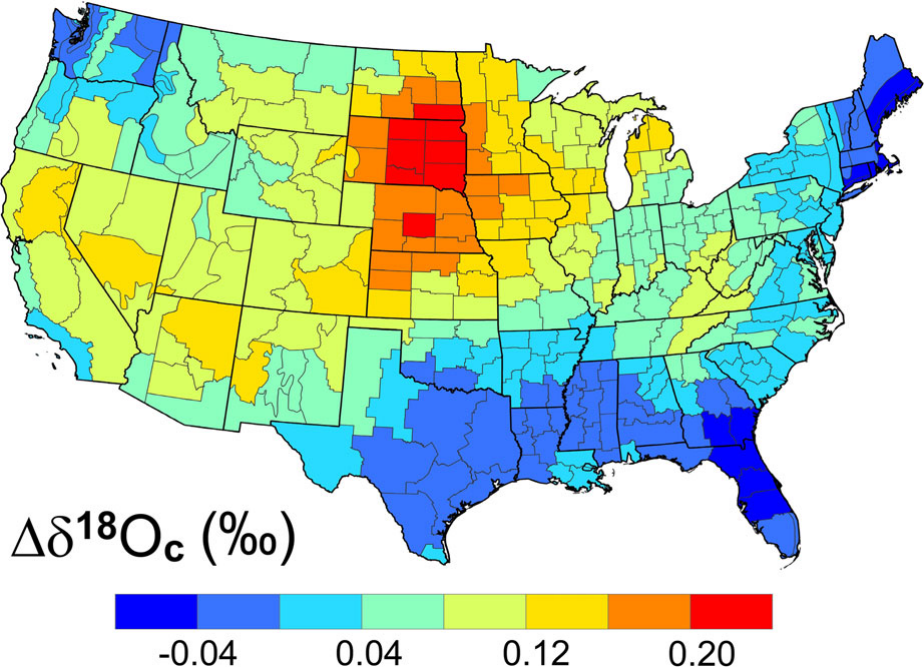
Isoscape of the predicted climate-driven shift in the δ^18^O_c(VPDB)_ (Δδ^18^O_c(VPDB)_) of the United States Air Force Academy (USAFA) cadets’ mid-root dentin (ca. 2002) compared to their crown enamel (ca. 1996).

The predicted Δδ^18^O_c_ isoscape for dentin versus enamel based on the NOAA/NCEI Climate Division temperature anomalies with Bayesian weighting is shown in Figure 6. It shows the predicted spatial distribution of the shifts in the δ^18^O_c_ values (Δδ^18^O_c_) between the cadets’ crown enamel and mid-root dentin. It assumed a constant +0.5 ‰/°C for the conversion of Δ°C anomalies to Δδ^18^O_c_ values and ignored factors explored by Kennedy et al. [13], such as decoupling of imbibed water from local surface waters.

Figure 6 represents paired, predicted Δδ^18^O_c_ data for the same teeth at the same location. It is important to note that the Δδ^18^O_c_ variation in Figure 6 for mid-root dentin versus enamel is due only to climatic fluctuations. The average year-to-year variations (±0.66 °C) in temperature anomalies for the conterminous USA due to shifting climatic patterns over the time of this study are more important than the small change in δ^18^O_c_ predicted by the 0.04 °C/year rise in temperatures between 1980 and 2005, even with the 5-year integration intervals used for amelogenesis and dentinogenesis.

Using 5-year intervals for integrating the climatic anomalies gives a Δ°C for the 1996 to 2002 interval of 0.27 ± 0.40 °C (1 s), which yields a Δδ^18^O_c_ = 0.063 ± 0.094‰ (1 s) using Equations (3) and (4). This means the differences are insignificant compared to interpolation (ca. ±1.5‰) and sampling (±0.5‰) errors. However, if a large population of individuals are averaged together (decreasing the standard error), or if the 5-year integration interval is narrowed to a half year (e.g. to the seasonal variation recorded in scalp hair), the Δ°C contribution to δ^18^O_c_ may become significant.

Relocating and temperature fluctuations are not expected to impact δ^18^O_c_ values until after age 8 when M3 amelogenesis begins. Surprisingly, a significant relationship was found between the measured and estimated Δδ^18^O_c_ values (Supporting Information Table S2) for the 26 cadets who had relocated by age 8: Δδ^18^O_c_ = 0.18(±0.13) + 0.076(±0.025)×Δδ^18^O_loc_ + 0.04(±0.31)×Δ°C (*R*^2^ = 0.26; *P* = 0.019; *df* = 26; ±1SE). This implies M3 mineralisation includes material incorporated or remobilised from before amelogenesis begins, blurring the tooth development times often used to calculate life trajectories. To a lesser extent due to its lower mobility, this also occurs with Pb [37]. Because the change in locations was significant, it may be that shifts in regional climatic patterns may also be significant, although it is expected to be a much smaller effect. The complex modelling required to separate these variables, given the multiple natal and relocation years, locations and climatic patterns, was not justified for the limited data set available for this study.

To put the magnitude of the predicted Δδ^18^O_c_ isoscape for the USAFA cadet population in Figure 6 in perspective, recently Chesson et al. [40] conducted matched analyses of 30 of the USAFA cadet teeth and found almost constant offsets of up to a 0.79‰ δ^18^O due to using 20 °C instead of 90 °C H_3_PO_4_ for the release of CO_2_ used in the IRMS measurements, and one-point instead of two-point normalisation during the analysis. These methodological offsets primarily affect the intercepts and not the slopes of conversion equations, but both will play a role when determining geolocations and trajectories. As with the Δ°C effect explored in this study, the difference is not large enough to change most interpretations with respect to geolocation, but the offsets are large enough that users of δ^18^O_c_ isoscapes should be aware of them. They are also large enough that the Δ°C effect will not be detectable unless the methods used for same-tooth enamel and dentin pair analyses are reproduced exactly.

To summarize, the observed δ^18^O_c_ and ^206^Pb/^207^Pb values from tooth enamel largely fit the expected isoscapes based on the reported birth locations [4,53]. Small differences between enamel and dentin are expected depending on factors such as the source of incorporated Pb, climatic patterns in the year the bioapatite is formed, the analysis method, which tooth is analysed, metabolic differences between individuals, differences in cooking methods, food choices and the balance of imbibed local water versus imported beverages. As Kennedy et al. [13] have noted, tap water sources are increasingly using groundwater and are being drawn from wider areas, so local precipitation can have different δ^18^O_w_ values than ingested tap water. With escalating urbanisation of populations, more people are drinking tap water (or bottled water) that is different from local precipitation sources. A similar shift is expected in the water contribution to incorporated Pb and ^206^Pb/^207^Pb. Furthermore, the enamel and dentin differences are more pronounced for Pb and its isotopes than for δ^18^O_c_, so there may be more to be gained from analysing both tissues for their Pb components than for δ^18^O_c_.

Finally, the Δ isoscapes demonstrate that O and Pb bioapatite isoscapes are not static, as climatic patterns, changes in metal sources, urbanisation of water sources and globalisation of food sources are tracked in the plotted values. This is apparent in the average decrease (ca. 0.1% of the range over the 6-year interval) found for the ^206^Pb/^207^Pb values in the USAFA cadet population compared to a similar increase found in the 1990s for crown enamel versus mid-root dentine in teeth from Scotland by Farmer et al. [54]. The difference in direction of the trends is likely due to Pb with ^206^Pb/^207^Pb = 1.04 from Australia being used in the UK *versus* ^206^Pb/^207^Pb = 1.22 from Missouri being used in the USA. Both are trending towards the world average (ca. 1.19 [11]). While these Δ isoscapes are small compared to the geolocation variation, they provide insight into the stasis and uncertainties of these isotope ratios as well as additional forensic information that may be helpful with geolocation of individuals.

## Conclusion

Firstly, the Δ plots of Pb highlight spatial changes in Pb over time. In addition to their use for determining life trajectories, they are useful for seeing the impact of environmental remediation and changes in Pb exposure in different region of the USA.

Secondly, a combination of isoscapes can help detect outliers that sometimes arise in one, but not multiple, isoscapes. They also provide additional information that may be used for narrowing the geolocation of individuals or the sourcing of contaminants.

Thirdly, the Pb and O tooth bioapatite isoscapes are not static. Based on long-term data in Keller et al. [4], environmental Pb isoscapes have been exponentially trending to global means in the USA at a rate of about 0.02%–0.05%/year (relative to their range of values), possibly due to a redistribution of dust globally as well as the increasing production of recycled Pb in secondary smelters. Tooth bioapatite tracks these environmental changes.

Lastly, the Δ plots, when coupled with the primary isoscapes, can improve the usefulness of the isoscapes, especially for fast-growing tissues. They may also shed light on the source of any measured differences in tissues formed at different times.
